# Evaluation of Emamectin Benzoate and Propiconazole for Management of a New Invasive Shot Hole Borer (*Euwallacea* nr. *fornicatus*, Coleoptera: Curculionidae) and Symbiotic Fungi in California Sycamores

**DOI:** 10.1093/jee/toy423

**Published:** 2019-01-12

**Authors:** Donald M Grosman, Akif Eskalen, Cavell Brownie

**Affiliations:** 1 Arborjet, Inc., Woburn; 2 Department of Plant Pathology, University of California, Davis, Davis; 3 Department of Statistics, North Carolina State University, Raleigh

**Keywords:** ambrosia beetle, chemical control, polyphagous shot hole borer, tree injection

## Abstract

The polyphagous shot hole borer (*Euwallacea* nr. *fornicatus*, Coleoptera: Curculionidae: Scolytinae), an exotic and invasive ambrosia beetle, was recently found attacking a number of tree species in Los Angeles, Orange, Riverside, and San Diego Counties in southern California. Their colonization and subsequent inoculation of a suite of symbiotic fungi that cause Fusarium Dieback, has resulted in extensive mortality of some tree species, including, California sycamore (*Platanus racemose* Nutt.). There are no sustainable control options for polyphagous shot hole borer other than maintaining tree vigor and removal of severely infested host material. The effectiveness of therapeutic treatments of an injected systemic insecticide containing emamectin benzoate (EB) alone and in combination with a systemic fungicide, propiconazole (P), was evaluated over a 4-yr period for maintaining the health of individual sycamore trees infested by polyphagous shot hole borer. All treatments containing EB reduced levels of polyphagous shot hole borer colonization and associated sap flow at attack sites compared to untreated controls. A second trial evaluated preventative treatments of EB and P alone or combined to protect individual sycamore from colonization by polyphagous shot hole borer. After 45 mo posttreatment, all treatments significantly reduced polyphagous shot hole borer attack levels and successful attacks compared to untreated controls (EB + P > EB alone > P alone). We concluded that EB alone or combined with P are acceptable therapeutic and preventative treatments for management of polyphagous shot hole borer in California sycamore in southern California.

The polyphagous shot hole borer, *Euwallacea* sp. nr. *fornicatus* (Coleoptera: Curculionidae: Scolytinae), is an ambrosia beetle native to Asia that was first introduced into the United States in Los Angeles County, California (Eskalen and Stouthamer 2012; Eskalen et al. 2012a,b, 2013). Since its discovery in 2003, polyphagous shot hole borer has spread to Orange, Riverside, San Bernardino, Ventura, and San Diego counties (Eskalen 2018, https://ucanr.edu/sites/pshb/).

Adult female beetles bore into trees to construct galleries in the sapwood where they inoculate symbiotic fungi, including *Fusarium euwallaceae*, *Graphium euwallaceae*, and *Paracremonium pembeum* (Freeman et al. 2013, 2016; Lynch et al. 2016). The former two species of fungi are the sole nutritional source for the adults and larvae. Unfortunately, for host plants, the *Fusarium* fungus is moderately pathogenic and disrupts water and nutrient movement within the vascular system causing the disease Fusarium Dieback (FD; Eskalen 2012, Eskalen and Stouthamer 2012). High levels of beetle infestation and fungal infection have resulted in high levels of tree mortality (Umeda 2017). Symptoms of beetle attack and FD vary by host species. The construction of tunnels by the beetles into the host’s sapwood severs vascular transport vessels resulting in variety of symptoms including wet staining (e.g., sycamore [*Platanus* sp.], maple [*Acer* sp.], willow [*Salix* sp.]), white powdery exudate (e.g., avocado [*Persea* sp.]), gumming (e.g., acacia [*Acacia* sp.], mimosa [*Albizia* sp.]), and/or frass (e.g., willow, maple) on the outer bark surface (Eskalen et al. 2017). While there is no visible injury to the bark at this stage of colonization, examination of the cortex and wood under the infested spot bored by the beetle reveals brown discolored necrosis caused by the fungus.

The currently recognized host range for the beetle–fungus complex includes more than 300 tree species that have been attacked by the beetle, of which more than 100 species can support growth of the fungus (Eskalen 2012, Eskalen et al. 2013). However, currently only 64 species are classified as reproductive hosts: that supports both beetle reproduction and associated fungal growth that causes FD (Eskalen 2018). These reproductive hosts include 18 tree species native to the United States (e.g., California sycamore [*Platanus racemosa* Nutt.], red and arroyo willows [*Salix laevigata* Bebb and *S. lasiolepis* Benth.], coast live and Engelmann oaks [*Quercus agrifolia* Née and *Q. engelmannii* Greene], and box elder [*Acer negundo* L.]), common urban landscape species (e.g., Japanese maple (*Acer palmatum* Thumb.) and sweetgum (*Liquidambar styaciflua* L.)), and an important agricultural commodity avocado (*Persea americana* Mill)).

Due to the polyphagous shot hole borer/Fusarium complex’s large host range, dieback and tree mortality is occurring in many landscapes—residential neighborhoods, parks, urban greenways, riparian areas, natural forests, and agricultural areas. Management tools are needed to reduce the economic and ecological impacts. Currently, management of the polyphagous shot hole borer/Fusarium complex is largely focused on cultural practices such as removal of dead and dying trees and sanitation of infested trees using solarization or chipping (Eatough-Jones and Paine 2015), and to a lesser extent, direct control using contact insecticides. Reding et al. (2013) and Eatough-Jones and Paine (2017) have shown that permethrin and bifenthrin sprays reduced ambrosia beetle attacks on host trees for 4 and 8 wk, respectively, after treatment.

A number of attempts had been made to evaluate systemic insecticides as safer alternatives to insecticide bole sprays in conifer forests with limited success (Fettig et al. 2013). However, more recently, emamectin benzoate (Syngenta Crop Protection), a phloem-mobile active ingredient was found capable of protecting loblolly pine (*Pinus taeda* L.) from colonization by *Ips* engravers beetles (Grosman and Upton 2006), ponderosa pine (*P. ponderosa* Dougl. ex Laws.) from mortality attributed to western pine beetle (*Dendroctonus brevicomis* LeConte) for three field seasons in California (Grosman et al. 2010), and green ash (*Fraxinus pennsylvanica* Marsh) from emerald ash borer (*Agrilus planipennis* Fairmaire) for 2–4 yr as well (Smitley et al. 2011). This and other research led to registration of a commercial formulation of emamectin benzoate for tree protection (TREE-äge; 4% [wt/wt] emamectin benzoate, Syngenta Crop Protection, Greensboro, NC) in 2010. Later, TREE-äge was demonstrated effective for protecting lodgepole pine (*P. contorta* Dougl. ex Loud.) from mortality attributed to mountain pine beetle (*D*. *ponderosae* Hopkins; Fettig et al. 2014) and Engelmann spruce (*Picea engelmannii* Parry ex. Engelm.) from mortality attributed to spruce beetle (*D. rufipennis* Kirby; Fettig et al. 2017). Eatough-Jones et al. (2017) and Mayorquin et al. (2018) found that treatments containing TREE-äge were effective in reducing polyphagous shot hole borer attacks on California sycamore, but evaluations only lasted for 6 and 12 mo, respectively. Longer term trials are needed to determine the duration of treatment efficacy.

As ambrosia beetles are reliant on their symbiotic fungi for survival, targeting the beetle’s food source is also a potential means for reducing the success of the beetle/disease complex within the host. Freeman et al. (2012) and Eskalen et al. (2016) have both evaluated several systemic fungicides in laboratory bioassays. One of the more effective active ingredients for inhibiting *Fusarium* sp. growth was propiconazole, a triazole fungicide. Propiconazole, applied via trunk injection, has shown efficacy against several beetle-vectored fungal diseases including oak wilt, Dutch elm disease, and laurel wilt disease (Appel and Kurdyla 1992, Stipes 1994, Eggers et al. 2005, Mayfield et al. 2008).

Currently, there are no known sustainable insecticide treatments with residual activity long enough to keep polyphagous shot hole borer from infesting trees for >90 d and no known suitable fungicide treatment that would inhibit growth of the fungus in the tree and in that way prevent beetle reproduction. In this study, we examined the efficacy of systemic injections of emamectin benzoate alone and combined with propiconazole as therapeutic and preventative treatments of California sycamores against polyphagous shot hole borer and its associated fungi.

## Materials and Methods

### Therapeutic Trial

This trial was conducted within the community of Pasadena Glen, Pasadena, CA (Los Angeles Co., about 37° 26 N, 122° 05 W, elev. ~183 m.). A survey was conducted in March 2013 of the location and general health of California sycamore within this community. From over 100 sycamores, thirty (30) relatively healthy-appearing trees, but already under some level of polyphagous shot hole borer attack, were randomly selected. Ten trees each were assigned to one of two injection treatments. The remaining 10 trees served as untreated controls. Test trees were spaced >2 m apart, and ranged 16– 91 cm diameter at breast height (DBH, ~1.4 m above ground level). Group assignments were controlled to confirm that there were no significant differences in DBH and initial number of polyphagous shot hole borer attacks from ground level to 1.8 m on the North-facing side of the stem.

There were three treatments: emamectin benzoate (EB, TREE-äge) only (treatment **1**); EB + propiconazole (P, Propizol, 14.3% [wt/wt] propiconazole, Arborjet Inc., Woburn, MA; treatment **2**); and untreated controls (treatment **3**). Each insecticide/ fungicide treatment (treatments 1–2) was injected at the labeled rate (2.0 ml TREE-äge or 2.4 ml Propizol per cm DBH) with the Arborjet QUIK-jet micro-injection system (Arborjet, Inc. Woburn, MA) into evenly spaced points (number is calculated by DBH/1.25) 0.3 m above the ground. Initial injections were made in March 2013. Each treatment was reapplied in March 2015.

Trees were evaluated for stem and crown condition at 1–6 mo intervals through the study period (49 mo). Each tree stem and crown were given a rating of 0 (healthy), 1 (polyphagous shot hole borer and *Fusarium* symptoms comprising <20% of the crown or dry stem), 2 (polyphagous shot hole borer and *Fusarium* symptoms comprising 20–80% of the crown or moist stem), 3 (polyphagous shot hole borer and *Fusarium* symptoms comprising >80% of the crown or wet stem; Mayfield et al. 2008), or 4 (dead tree). At the same time, the number of new polyphagous shot hole borer attacks and *Fusarium*-caused bleeding wounds was counted and marked (using different colored paint pens) from ground level to 1.8 m on the North-facing side of the stem. The North side was used because initial attacks by the beetle tended to target this area of the bole (D.G., personal observations), presumably because this area tends to have more constant diurnal temperatures which favor both beetle and fungal symbionts colonization success. Length of count area and DBH/4 was used to calculate surface area monitored for each tree. Initial attack level and subsequent attacks per sample period was calculated as attacks per m^2^. Also, in October 2013 and December 2014, the level of bleeding/sap flow from polyphagous shot hole borer attacks on the bark surface of each tree was ranked as 1 (dry, very little or no sap flow), 2 (moist, moderate sap flow), or 3 (wet, extensive sap flow).

### Statistical Analyses

A one-way analysis of variance (ANOVA) was applied to each date to measurements collected at a limited number of dates. For new attacks per m^2^, which was measured on 13 dates, a repeated measures (RM) ANOVA was performed using SAS Proc Mixed (SAS Institute 2008). Several error covariance structures were compared for carrying out the RM ANOVA on cube root transformed new attacks per m^2^ (*Y* = [X + 0.5]^1/3^) for the 13 measurement dates and a final model was selected based on the Bayesian Information Criterion (BIC, Schwarz 1978). The chosen mixed model included Treatment, Date and interaction as fixed effects and tree within Treatment as random. In addition, the error covariance selected was a generalization of compound symmetry having constant between-date correlations and variance dependent on date, implemented using a Repeated statement with option TYPE = CSH, and denominator degrees of freedom (df) obtained using the Kenward-Roger method via option DDFM = KR (Kenward and Roger 1997). An LSmeans statement with options PDIFF and SLICE = Date was used to obtain means for all Treatment by Date combinations and to test for differences between treatments at each Date. The comparisons of each injection treatment to the untreated control at each date were considered *a priori* comparisons and performed using a comparison-wise significance level of 0.5 (which is equivalent to performing the selected comparisons using the LSD procedure).

### Preventative Trial

This study was conducted at three locations: on the California Institute of Technology (Cal Tech) campus (Pasadena, CA; about 34°14 N, 118°12 W, elev. 207 m), a portion of the Rose Bowl parking lot (Pasadena, CA; about 34°15 N, 118°17 W, elev. 253 m), and the Fairplex and Sheraton Hotel parking lot (Pomona, CA; about 34°04 N, 117°45 W, elev. 295 m). A survey was conducted in January 2014 of these locations and general healths of California sycamores were determined. Only uninfested and lightly (≤5 polyphagous shot hole borer attacks overall) infested trees were included in the trial. At Cal Tech and Fairplex, 80 trees were selected, while 36 were selected at Rose Bowl. Each of three injection treatments was randomly assigned to 20 trees (Cal Tech and Fairplex) or nine trees (Rose Bowl). An equal number of trees served as untreated controls at each site.

There were four treatments: 1) emamectin benzoate (EB; TREE-äge) only; 2) propiconazole (P, Propizol) only; 3) EB + P combination treatment; and 4) untreated controls. Each insecticide/ fungicide treatment (treatments 1–3) was applied at the same rate and manner as described for the therapeutic trial. Injections were applied only once in January 2014.

Study trees were evaluated for stem and crown condition approximately every six months for 45 mo. Each tree stem and crown was given a health rating as described above. The number of polyphagous shot hole borer attacks and *Fusarium*-caused bleeding wounds were counted and marked with paint pens from ground level to 1.8 m on the North facing side of the stem.

### Statistical Analyses

Measurements collected at a single date were subjected to two-way ANOVA with fixed effects Treatment, Site and Treatment by Site interaction. An RM ANOVA was carried out on log-transformed new attacks per m^2^ (*Y* = log[*X* + 1]) from nine measurement dates at each of three sites, using the Proc Mixed procedure of SAS (SAS Institute 2008). The mixed model included Treatment, Site, Date and all interactions as fixed effects. Based on the BIC, the error covariance was again a generalization of compound symmetry implemented using a Repeated statement with option TYPE = CSH. Denominator df were obtained via option DDFM = KR. Similar to the Therapeutic trial, results of the RM ANOVA indicated that attack levels differed strongly by date. Therefore, to examine treatment effects separately under differing attack levels, An LSmeans statement with options PDIFF and SLICE = Date was used to compare treatments averaged over sites, and separated by date, using comparison-wise significance level of 0.05 to compare each of the three injection treatment to the untreated control at the same date.

A split-plot type of RM analysis was carried out on log-transformed condition scores from two measurement dates at each of the three sites using Proc Mixed. Treatment, Site, Date and all interactions were fixed effects, and Tree within Treatment and Site, or ‘whole plot error,’ was included as a random effect. Comparisons of each injection treatment to the untreated control were carried out using a comparison-wise significance level α = 0.05 on means over dates and sites provided interactions between these factors and treatment were not significant

## Results

### Therapeutic Trial

**Fig. 1.**
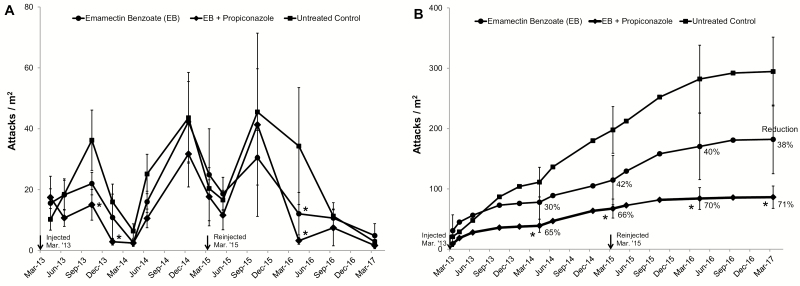
Mean (±SE) number of new (A) and cumulative (B) polyphagous shot hole borer attacks per m^2^ on California sycamores 1–49 mo after therapeutic systemic pesticide treatment at Pasadena Glen, Pasadena CA. * indicates that on a given date the treatment mean is significantly different from the untreated control mean (LSD, α = 0.05).

During this trial, one tree (treated with emamectin benzoate alone) was removed in late 2014 due to hazard concerns; therefore the data collected from this tree prior to removal was excluded from data analysis. Of the remaining trees, the mean DBH (48–56 cm) and initial polyphagous shot hole borer attack level (21–28/m^2^) did not differ among treatments (*F* = 0.62; df = 2, 28; *P* = 0.546, *F*= 0.04; df = 2,28; *P* = 0.962, respectively). Results from the RM analysis of cube root transformed new attacks/m^2^ showed highly significant date effects (*F* = 20.86; df = 12, *96; P* < 0.001) but differences between treatment groups, averaged over dates, and the treatment by date interaction, were not significant (*F* = 2.07; df = 2, 26.6; *P* = 0.146 and *F* = 1.03; df = 24, 128; *P* = 0.433, respectively). Examination of mean attacks/m^2^ at each monitoring period indicated that the initial gain through June 2013 did not differ significantly among treatment groups (Fig. 1A). On nearly all other subsequent evaluations beetle attacks on untreated controls exceeded attacks on injected trees. Over time, it was apparent that the highly significant Date effect was associated with markedly higher polyphagous shot hole borer attack levels during warmer months (April–November) and treatment comparisons by date indicates that attacks were significantly fewer on EB + P trees compared to untreated controls for the count intervals ending in October 2013 (*t* = −2.33; df = 34.1; *P* = 0.026), January 2014 (*t* = −2.21; df = 29.4; *P* = 0.035), and April 2016 (*t* = −2.82; df = 25.6; *P* = 0.0092). After June 2013, attacks on EB only trees were always numerically lower than attacks on controls, but only significantly lower on the evaluation done in April 2016 (*t* = −2.10; df = 25.6; *P* = 0.045). Figure 1B shows the mean cumulative number of polyphagous shot hole borer attacks for each treatment over the 49 mo period. By the end of the trial, attacks ranged from 86 to 294/m^2^. Overall, EB alone and EB + P reduced polyphagous shot hole borer attacks by 38% and 71%, respectively, compared to untreated controls, but only EB + P reductions were significant at each annual evaluation.

**Fig. 2.**
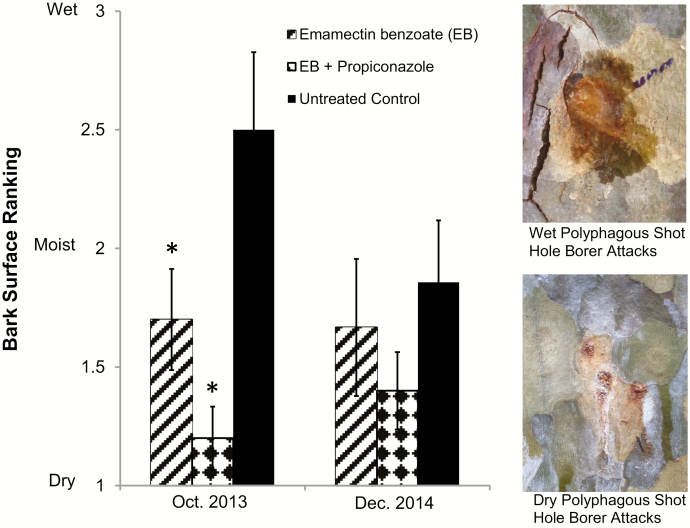
Mean (±SE) rank of stem condition on California sycamore 7 and 24 mo after therapeutic systemic pesticide treatment. Condition: 1 = dry, 2 = moist, and 3 = wet. *Treatment mean is significantly different from untreated control mean (LSD, α = 0.05).

The level of bleeding/sap flow from polyphagous shot hole borer attacks on the stem of each tree was significantly lower (drier; *F* = 7.05; df = 2, 26; *P* = 0.004) for both injection treatments compared to untreated controls in October 2013 (Fig. 2). In addition, trees treated with EB + P treatment were drier than EB alone. The drying of the main stem was very evident on the EB + P treatment trees. The level of bleeding in December 2014 showed a similar trend but did not differ among treatments (*f* = 0.89; df = 2, 25; *P* = 0.424).

The overall condition score of most trees was very good (0.08– 0.30) at the beginning of the study with relatively little evidence of dieback and no differences among the treatments (*F* = 0.79; df = 2, 28; *P* = 0.464; Table 1). However, several untreated control trees began to decline rapidly over the next year to the extent that two trees died and the mean rank became significantly worse compared to EB + P trees by December 2014 (*F* = 4.06; df = 2, 28; *P* = 0.029). The condition of EB alone trees scored better but were not significantly different from the controls. By April 2016, one tree from each treatment had died; however, the overall scores of the EB + P treatment declined at a slower rate compared to the controls and EB only treatments (*F* = 1.88; df = 2, 28; *P* = 0.172). By March 2017, only 6 of 10 (60%) untreated control trees had survived (Table 1). In contrast, 7 of 9 (77.8%) EB alone treated trees and 9 of 10 (90%) EB + P trees survived.

**Table 1.** Mean (±SE) tree condition and mortality of California sycamore (CS) over time at Pasadena Glen (PG), Pasadena, CA: 2013–2017

		Initial (Mar. 2013)		Dec. 2014		Apr. 2016		Mar. 2017	
Treatment	*N*	Condition	Pct. mortality	Condition	Pct. mortality	Condition	Pct. mortality	Condition	Pct. mortality
Emamectin benzoate (EB) alone	9	0.15 ± 0.11	0.0	0.39 ± 0.20	0.0	1.28 ± 0.49	11.1	1.72 ± 0.59	22.2
EB ± propiconazole	10	0.07 ± 0.07	0.0	0.15 ± 0.15*	0.0	0.60 ± 0.40*	10.0	0.60 ± 0.41*	10.0
Untreated control	10	0.30 ± 0.21	0.0	1.20 ± 0.51	20.0	1.82 ± 0.56	30.0	2.75 ± 0.57	40.0
Remaining untreated CS in PG	51							2.71 ± 0.32	27.5

Tree condition ranking: 0 = healthy; 1 = polyphagous shot hole borer and *Fusarium* symptom comprising <20% of the crown or dry stem; 2 = polyphagous shot hole borer and *Fusarium* symptoms comprising 20–80% of the crown or moist stem; 3 = polyphagous shot hole borer and *Fusarium* symptoms comprising >80% of the crown or wet stem; 4 = Dead tree.

*Significant difference (*P* < 0.05) between systemic treatment Condition means and untreated control (LSD, α = 0.05).

### Preventative Trial

**Fig. 3.**
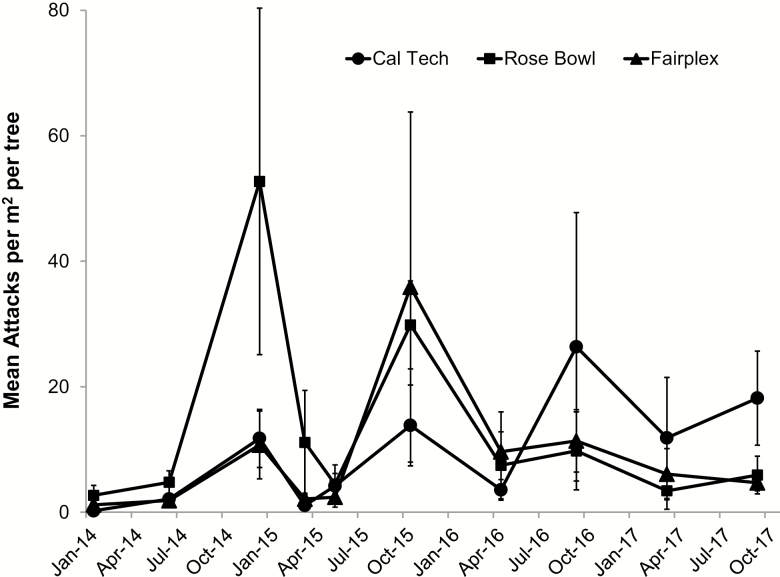
Mean (± SE) number of new polyphagous shot hole borer attacks per m^2^ on untreated California sycamores 2013–2017 at Rose Bowl and Cal Tech, Pasadena, and Fairplex, Pomona, CA.

**Fig. 4.**
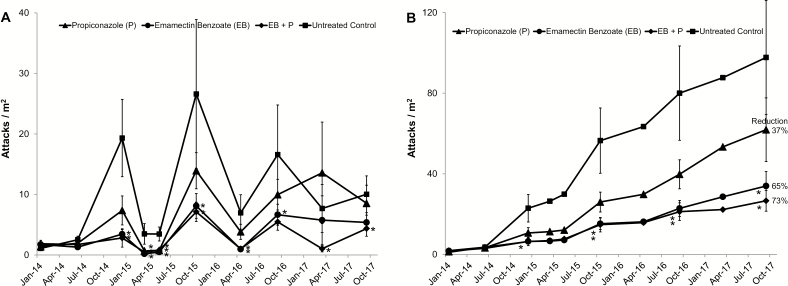
Mean (±SE) number of new (A) and cumulative (B) polyphagous shot hole borer attacks per m^2^ on California sycamores for treatments 0–45 mo after preventative systemic pesticide treatment. Means are averages over sites at Rose Bowl, Fairplex and Cal Tech and * indicates that treatment mean is significantly different from the untreated control mean (LSD, α = 0.05).

The three trial sites had similar tree species and condition, evaluation times, and did not differ significantly in the overall number of polyphagous shot hole borer attacks (*F* = 0.66; df = 2, 180; *P* = 0.519) and condition scores (*F* = 1.97; df = 2, 177; *P* = 0.142). As such, data were combined for analysis. As in the previous trial, the mean DBH and polyphagous shot hole borer attack level on study trees did not differ significantly (*F* = 0.29; df = 3, 190; *P* = 0.833, *F*= 1.02; df = 3, 190; *P* = 0.385, respectively) among treatment groups at the initiation of the study. All sites had similar fluctuations in attack levels on untreated controls each year (Fig. 3): generally increasing attack levels during periods of warmer weather (April– November), and declining attack levels during the cooler months (December–March) resulting in a highly significant Date main effect (*F* = 45.18; df = 8, 670; *P* < 0.001). There was also a significant main effect for treatments (*F* = 6.62; df = 3, 180; *P* < 0.001) and a Site by Date interaction (*F* = 4.87; df = 16, 877; *P* < 0.001), though none of the interactions with treatment were significant (*P* > 0.20 for all other effects). Averaging over Sites and Dates, attacks were significantly lower for treatments EB + P and EB alone compared to the untreated controls (t = 4.07, df = 180, *P* < 0.001 and *t* = 3.40, df = 180; *P* < 0.001, respectively), but not for *P* alone (*P* = 0.088). However, treatment effects varied by Date and averaged over Site (Fig. 4A).

Figure 4B shows the mean cumulative number of polyphagous shot hole borer attacks for each treatment over the 45-mo period. The slope of cumulative attacks for each treatment varied over time. During the initial 5 mo after trial initiation, attack levels were similar among treatments. Thereafter, however, attacks on untreated control trees accumulated at a faster rate. In contrast, the accumulation of attacks on EB + P treated trees was significantly slower than for checks at least to December 2014 (*F* = 3.33; df = 190; *P* = 0.021) and for both EB and EB + P as of October 2015, Sep. 2016 and Sep. 2017 (*F* = 4.86; df = 190; *P* = 0.003, *F* = 5.42; df = 190; *P* = 0.001, *F* = 6.20; df = 190; *P* > 0.001, respectively). The observed accumulation of attacks on EB and EB + P treated trees was remarkably similar until the last two evaluation periods. EB + P trees maintained the same slow accumulation of attacks through the entire study period. Attacks on P alone trees were numerically lower, but not significantly different from those on check trees. The end point reduction in attacks compared to controls averaged 37% (range 22–52%) for P alone, 65% (range 56–84%) for EB alone, and 73% (range 64–81%) for EB + P.

**Fig. 5.**
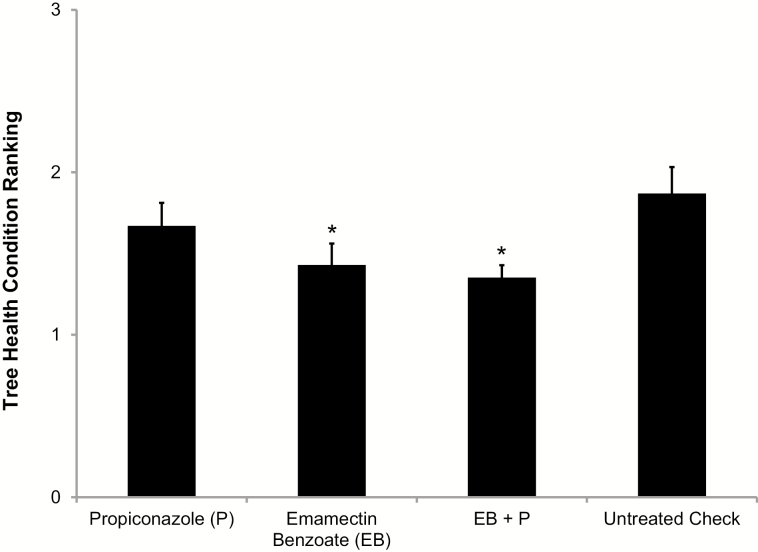
Mean (±SE) tree condition ranking of California sycamore 45 mo after preventative systemic pesticide treatment. * indicates that treatment mean is significantly different from the untreated control mean (LSD, α = 0.05).

As of September 2017, 45 mo posttreatment, the condition of trees treated with EB alone or combined with P, were in significantly better health compared to untreated controls (*F* = 3.50, df = 3, 72, *P* = 0.020) (Fig. 5). By the end of the trial, only one untreated control and P trees had died.

## Discussion

Overall, EB + P was the most effective treatment for reducing polyphagous shot hole borer attack levels, sap flow/bleeding from attack holes, proportion of beetle emergence and tree mortality. All treatments (EB + P > EB alone > P alone) reduced polyphagous shot hole borer attack levels, but preventative treatments were most effective. The duration of treatment efficacy was variable and appears to be influenced by polyphagous shot hole borer attack levels at the time of treatment. When attack levels are initially light (<5 attacks/m^2^) then P alone can reduce attacks for 12–18 mo before fading. EB alone appears to be more durable lasting for one year when beetle pressure is high or as many as 2+ yr when pressure is relatively light. EB + P had the greatest durability; being able to suppress beetle attacks for 2 yr under high beetle pressure and 4+ yr under light pressure.

All sites had at least some polyphagous shot hole borer attacks on untreated control trees throughout each year of the studies and also had similar fluctuations in attack levels each year: generally increasing attack levels during periods of warmer weather (generally April–November, when average high temperatures exceed 21C, Umeda 2017) and declining attack levels during the cooler months (December–March). Umeda (2017) calculated that most likely there are 3–6 overlapping generations of polyphagous shot hole borer per year in the Los Angeles County area. Because of the lack of synchrony of generations, management with timed spray application of contact insecticides is difficult. In addition, control of polyphagous shot hole borer with contact insecticides is difficult due to 1) their cryptic nature (i.e., >95% of time hidden inside the tree), 2) feed on fungi not on plants, 3) short life cycle (30–60 d), 4) high reproductive rates (40 progeny / generation), and 5) ability to attack trees throughout the year. The use of systemic pesticides with extended residual activities could alleviate some of these limitations.

Pasadena Glen sycamores had an average of 24 attacks/m^2^ at the beginning of the trial. By the end of year 2 (Mar 2015) the control trees averaged 197 attacks/m^2^, while EB alone and EB + P averaged 115 and 67 attacks/m^2^, respectively (Fig. 1B). The EB +P combination was the only treatment capable of significantly reducing beetle attacks during the first 2 yr by as much as 66%. Because of concern about continued accumulated attacks and associated fungal infections the researchers elected to retreat trees early in 2015 to try to improve if not maintain EB treatment efficacy for the next 2 yr. In response, efficacy of EB alone was maintained around 40%, while EB + P improved to 70% below controls by the end of the third year. By the end of the fourth year, EB efficacy was beginning to decline while EB + P remained relatively stable. This trial provides evidence of moderate efficacy of EB alone for reducing polyphagous shot hole borer attack levels for at least 24 mo under moderate to high beetle pressure. In contrast, under the same conditions, EB + P is able to reduce attack levels and tree mortality to a greater extent and for a longer period of time.

Although ambrosia beetle are not phloeophagous like bark beetles, it was demonstrated that redbay ambrosia beetle adults are susceptible to (i.e., killed by) EB treatment (Pena and Carrillo 2013). When injected systemically into trees, EB must be ingested by the target pest to have an effect. Thus, the adult polyphagous shot hole borer must be taking in some plant material as they tunnel through the phloem and xylem tissue during initial gallery construction. In addition, Freeman et al. (2012) determined that EB has some fungicidal activity; reducing fungal growth by 45%. Therefore, beetle mortality also could be due in part to starvation caused by reduced or absence of symbiotic fungi growth in the presence of EB.

The therapeutic treatments appear to have an indirect effect toward reducing bleeding/sap flow (EB + P > EB alone) thus reducing attraction of conspecifics to already infested trees. Tree sap chemistries and/or metabolites are known to function as kairomones that attract insects to their hosts. For example, several bark and ambrosia beetles are attracted to ethanol that is emitted from their hosts (Kelsey et al. 2013). Similar, quercivorol is a chemical released by *Fusarium* fungi and likely serves as an attractive kairomone to polyphagous shot hole borer (Byers et al. 2017, Carrillo et al. 2015, Cooperband et al. 2017). It is likely that reduction in attraction of polyphagous shot hole borer to treated trees is linked to a reduction in one or more chemicals that is normally released from infested trees.

Based on the above results, we recommend that emamectin benzoate alone can be applied as a preventative treatment, particularly with few (≤10 attacks) or no attacks on lower 2 m of trunk or when other attacked trees are within 50 m of a reproductive host. We recommend that the combination of emamectin benzoate and propiconazole be applied when the number of polyphagous shot hole borer attacks on a tree exceeds 10 on the lower 2 m of trunk as this treatment has more beneficial effect at reducing polyphagous shot hole borer/*Fusarium* success at all infestation levels. Retreatment is recommended at 2-yr intervals.

Unlike contact pesticide sprays that may provide some immediate protection, systemic pesticides require some period of time to move within the tree’s vascular system to target areas. The time necessary to complete chemical distribution and the extent of this movement depends on a number of factors including, host species vasculature, moisture availability, temperatures, and the extent of beetle infestation and fungal infection at the time of treatment. Overall, preventative treatments applied to healthy, uncompromised trees proved to be far more efficacious than therapeutic treatments. As such, we recommend that preventative treatments be made when new polyphagous shot hole borer attacks are found on neighboring trees within 50 m of the tree(s) of interest. Treatments can be made any time of year when there is good soil moisture to encourage uniform distribution throughout the tree. Trunk injections of insecticide alone or combined with fungicide will distribute upward in the tree within 4–6 wk with adequate soil moisture (D.G., personal observations).
